# Effects of DNA Methylation of HPA-Axis Genes of F1 Juvenile Induced by Maternal Density Stress on Behavior and Immune Traits in Root Voles (*Microtus oeconomus*)—A Field Experiment

**DOI:** 10.3390/ani14172467

**Published:** 2024-08-25

**Authors:** Shouyang Du, Guozhen Shang, Xin Tian, Zihan Liu, Yanbin Yang, Hongxing Niu, Jianghui Bian, Yan Wu, Jinyou Ma

**Affiliations:** 1 Postdoctoral Research Base, Henan Institute of Science and Technology, Xinxiang 453003, China; dsy862323@126.com; 2 College of Animal Science and Veterinary Medicine, Henan Institute of Science and Technology, Xinxiang 453003, China; 18336090210@163.com (X.T.); zihan18638736840@163.com (Z.L.); 3 Key Laboratory of Adaptation and Evolution of Plateau Biota, Northwest Institute of Plateau Biology, Chinese Academy of Sciences, Xining 810001, China; shangguozhen@nwipb.cas.cn (G.S.); bjh@nwipb.cas.cn (J.B.); 4 Qinghai Key Laboratory of Animal Ecological Genomics, Xining 810001, China; 5 College of Veterinary Medicine, Henan Agricultural University, Zhengzhou 450007, China; yangyb@henau.edu.cn; 6 College of Life Science, Henan Normal University, Xinxiang 453007, China; hongxingniu@htu.cn; 7 School of Life and Environment Sciences, Hangzhou Normal University, Hangzhou 310012, China

**Keywords:** maternal stress, population density, depressive and anxiety-like behaviors, immunocompetence, DNA methylation, root vole

## Abstract

**Simple Summary:**

It is widely accepted that maternal stress can influence the behavior and immunity of F1 in the laboratory. However, surprisingly little is known about the replicated studies on the mechanisms by which maternal stress drives individual characteristics in wild animals. Therefore, to study the possible effect of DNA methylation of hypothalamic-pituitary-adrenal-axis (HPA-axis) genes of F1 induced by maternal density stress on behavior and immune traits in root voles, we measured mRNA and protein expression and DNA methylation of HPA-axis genes and assessed the depressive and anxiety-like behaviors and the immune traits. The F1 from high density had a lower DNA methylation level of corticotropin-releasing hormone (CRH) and a higher DNA methylation level of glucocorticoid receptor gene (*NR3C1*), accompanied by an increase in the expression levels of the CRH mRNA and protein expression, and a reduction in the expression levels of the *NR3C1* mRNA and protein expression. The F1 from high density also reduced anti-keyhole limpet hemocyanin immunoglobulin G levels and phytohemagglutinin-delayed hypersensitivity, increased cytokines and depressive and anxiety-like behaviors, and increased coccidial infection. Our results indicate that altering the prenatal intrinsic stress environment can fundamentally impact behavior and immunity by DNA methylation of HPA-axis genes and can further drive population fluctuations in wild animals.

**Abstract:**

The literature shows that maternal stress can influence behavior and immune function in F1. Yet, most studies on these are from the laboratory, and replicated studies on the mechanisms by which maternal stress drives individual characteristics are still not fully understood in wild animals. We manipulated high- and low-density parental population density using large-scale field enclosures and examined behavior and immune traits. Within the field enclosures, we assessed anti-keyhole limpet hemocyanin immunoglobulin G (anti-KLH IgG) level, phytohemagglutinin (PHA) responses, hematology, cytokines, the depressive and anxiety-like behaviors and prevalence and intensity of coccidial infection. We then collected brain tissue from juvenile voles born at high or low density, quantified mRNA and protein expression of corticotropin-releasing hormone (CRH) and glucocorticoid receptor gene (*NR3C1*) and measured DNA methylation at CpG sites in a region that was highly conserved with the prairie vole CRH and *NR3C1* promoter. At high density, we found that the F1 had a lower DNA methylation level of CRH and a higher DNA methylation level of *NR3C1*, which resulted in an increase in the expression levels of the CRH mRNA and protein expression and further reduced the expression levels of the *NR3C1* mRNA and protein expression, and ultimately led to have delayed responses to acute immobilization stress. Juvenile voles born at high density also reduced anti-KLH IgG levels and PHA responses, increased cytokines, and depressive and anxiety-like behaviors, and the effects further led to higher coccidial infection. From the perspective of population density inducing the changes in behavior and immunity at the brain level, our results showed a physiological epigenetic mechanism for population self-regulation in voles. Our results indicate that altering the prenatal intrinsic stress environment can fundamentally impact behavior and immunity by DNA methylation of HPA-axis genes and can further drive population fluctuations in wild animals.

## 1. Introduction

In mammal populations, both extrinsic and intrinsic factors regulate population fluctuations under natural environments [1]. The stress caused by various environmental factors during pregnancy and the early postpartum period can have long-term effects on adult F1, mainly including reproduction, immunity, and behavior [2,3]. The maternal effect refers to the direct effect of the phenotype of both parents on the phenotype of F1, which plays an important role in population decline and maintenance of longer low-quantity stages [4]. Our previous research has shown that the maternal effect acts as an adaptive bridge in translating maternal environments into F1 phenotypes, thereby affecting population dynamics [5]. Population density has been considered to be a significant physiological stressor determining life history traits in a small mammal population [5]. Therefore, exploring the long-term effects of maternal density stress on F1 will be of great significance for population regulation.

The hypothalamus-pituitary-adrenal (HPA) axis is a known responder during stress, causing an elevation of corticosterone or cortisol, a glucocorticoid (GC) hormone that regulates endocrine and behavioral activities [6]. The hypothalamus can synthesize and release corticotrophin-releasing hormone (CRH). The secretion of CRH can activate the release of adrenocorticotropic hormone (ACTH) from the pituitary. Then the ACTH enters the blood and activates the adrenal gland to produce corticosterone or cortisol. The GC starts to bind to the glucocorticoid receptor (GR). The GR, which is closely related to the negative feedback function of HPA axes, is a polypeptide that binds GC with high affinity [6]. Maternal stress can affect the functional activity of the HPA axis by affecting the expression of hypothalamic CRH and hippocampal GR in F1 [7]. Phenotypic plasticity enables a single genotype to produce multiple morphological, behavioral, and physiological characteristics, which play an important role in adaptability and population dynamics [8]. Recent studies have demonstrated that DNA methylation can effectively encode social experiences, leading to enduring alterations in gene expression and the emergence of distinct phenotypic traits [9,10,11]. Maternal stress has long-term effects on the expression and DNA methylation of genes related to the HPA axis in F1 [10]. However, under maternal stress conditions, the studies on the expression and promoter methylation of related genes of the HPA axis in F1 mainly focus on laboratory animals and humans, while research on wild animals under natural conditions is still lacking.

Immunity is not just one of the very important factors of individual fitness [12] but also an important way of preventing pathogens from invading [13] and is important to the population dynamics in small mammals [14]. The immunity of mammals can determine the survival of animals, which is related to the functional characteristics of the F1 HPA axis [15]. Depression and anxiety, which can have negative health consequences, are complex mental illnesses, and their pathogenesis involves multiple aspects. These diseases are mainly caused by multiple factors in the body, including abnormalities in the HPA axis, damage to the limbic system, and imbalance of inflammatory cytokines [16,17]. However, the DNA methylation mechanism of genes related to the HPA axis that affects the immune function and depressive and anxiety-like behaviors of F1 in wild animals is currently unclear.

In the present study, we investigated the effects of DNA methylation of HPA-axis genes of F1 induced by maternal density stress on behavior and immune traits in root voles. Parental enclosures with high (30 voles per sex per enclosure) and low (6 voles per sex per enclosure) population densities were initially established to breed F1. The immune traits and depressive and anxiety-like behaviors in the two treatment groups were measured following live trapping in the breeding seasons in F1. We also evaluated the negative feedback function of the HPA axis, inflammatory cytokines, and the mRNA and protein expression and DNA methylation of HPA-axis genes in the F1. This study aimed to test whether maternal density stress affects immune traits and depressive and anxiety-like behaviors by altering DNA methylation modifications of HPA-axis genes in F1. We hypothesized that maternal density stress could alter the DNA methylation levels of HPA-axis genes in F1, reduce immunity, and increase depressive and anxiety-like behaviors.

## 2. Materials and Methods

### 2.1. Root Voles in the Study Area

The study was conducted at Haibei Alpine Meadow Ecosystem Research Station, Menyuan County, approximately 155 km north of Xining, the capital city of Qinghai Province, People’s Republic of China (37°37′ N, 101°12′ E). The predominant plants of the study site were *E. nutans*, *Poa* sp., *Kobresia humilis*, *Carex* spp., and *Potentilla fruticosa*, the natural habitat of root voles. Root vole populations in this area fluctuate only seasonally, with the lowest levels occurring in early spring; multiyear cycles are weak or absent [18]. The average population size in the habitat ranged from 217 to 280 voles ha^−1^ in autumn, where grazing activities were limited; where vegetation consisted mainly of *E. nutans*, the population density was approximately 400 voles ha^−1^ in autumn [19]. The breeding season typically lasts from April to late October. Juveniles reach puberty and breeding age at approximately 50 and 70 days, respectively.

### 2.2. Experimental Facility

The experiment was conducted in eight 0.15 ha (50 × 30 m) outdoor enclosures, which used galvanized steel panels (1.5 m above and 0.5 m below ground) in order to prevent mammalian predators from gaining entry. In each enclosure, Avian predators were prevented from entering through a 3 × 3 cm grid wire mesh held aloft by a central pillar (10 × 250 cm). Each enclosure was equipped with 60 laboratory-made wooden traps [5] spaced in 5 × 5 m grids. The wooden sheet was covered in each trap to protect root voles from exposure to precipitation and extreme temperatures.

### 2.3. Establishment of Parental Populations and Live-Trapping

In brief, a total of 72 voles for each sex, 6 months of age or older, were used to establish parental populations. They were either F2 generations born in the laboratory or captured as juveniles from the previous year. All individuals were earmarked before they were released into the enclosures. The initial body weights (*p* = 0.781) did not differ among voles in different enclosures. We established parental populations in four enclosures in mid-June 2019 at two density conditions. The high-density population consisted of 30 adults of each sex into each of two enclosures, and low-density consisted of 6 adults of each sex into another two enclosures, representing high and moderate densities, respectively [18]. After allowing the animals to acclimate to their new environments for approximately 2 weeks, we started live-trapping on 20 June 2019 and lasted until 29 August 2019. Standard capture–record–recapture methods were used throughout the whole study. Five trapping sessions were conducted, and each trapping session comprised 3 trapping days. The time intervals between two trapping sessions were approximately 1 week. The traps were set between 7:00 a.m. and 7:00 p.m., checked every 2 h, and the locks closed when trapping did not occur. Following each capture, we recorded animal identification, sex, body mass, and reproductive status (males, testes abdominal or scrotal; females, palpable embryos, and enlarged teats barren of hair), and then the animal was released back to the enclosures. F1 were captured at 20–30 days of age (10–16 g) [5,20] and then were removed to the laboratory for use in subsequent experiments.

### 2.4. Behavioral Tests

When the F1 were captured in the laboratory, they were maintained in the temperature-controlled colony (20 ± 2 °C) and housed singly in plastic cages with sawdust and with pellet chow and water available ad libitum until the subsequent experiments. A well-trained investigator conducted the behavioral tests, including elevated plus maze (EPM) and the open field test (OFT) as described previously [21]. Behavioral testing was carried out in the same order for all voles at 08:30–12:30 h.

The EPM device, 55 cm above the ground, consisted of two open arms (35 × 5 cm), two closed arms (35 × 5 × 15 cm), and a central area (5 × 5 cm). The voles were placed in the central square facing an open arm and allowed to explore for 5 min while using Panlab SMART 3.0 video tracking software recorded their behavior. The distance and time in open arms, entries in open arms, and total distance were analyzed [21]. After each test, the bottom of the open field was also thoroughly cleaned with 75% ethanol to prevent residual odors. The falling animal was removed from the study.

Voles were placed in the center of the arena (50 × 50 × 15 cm) and allowed to explore the open field for 5 min. Then the distance and time in the center zone, total rest time, and total distance were recorded using Panlab SMART 3.0 video tracking software [21]. The floor surface and the inner wall of the chamber were thoroughly cleaned with 75% ethanol to eliminate any odors after each test.

### 2.5. Fecal and Blood Samples Preparation

Fecal samples for measuring the fecal corticosterone metabolite (FCM) of 0 (starting from the third trapping period) were collected once during the first 2 h of trapping (07:00–09:00) from each enclosure during each trapping session. Thus, samples were not contaminated by any other intruders. Fecal samples for the infection status of coccidia parasites were collected when the F1 were first captured in the laboratory. Collected samples were respectively frozen at −20 °C and refrigerated at 4–8 °C until analysis.

The acute stress responses of different treated F1 were tested. We used transparent plastic cylinders (3 cm in diameter and 10 cm long) to conduct an acute immobilization stress for 2 h at 4:00 p.m. Fecal samples were respectively collected at 0 (before the acute immobilization stress), 2 (at the end of stress), 14, 20, 26, 32, and 38 h, and stored at −20 °C until analysis.

The blood sample was collected from the retro-orbital vein of the captured F1 to test leukocyte counts. After 2 days, cell-mediated immunity was measured by phytohemagglutinin (PHA) assay [15]. Four days later, these F1 individuals received a single subcutaneous injection of 0.15 mg of the novel antigen KLH suspended in 0.1 mL sterile saline. After ten days, the blood samples were collected (50–100 μL) from the retro-orbital sinus to measure anti-keyhole limpet hemocyanin immunoglobulin G (anti-KLH IgG) levels in those injected F1. Blood samples were centrifuged at 4 °C for 15 min at 4000 rpm, and serum was collected and stored at −80 °C until analysis.

### 2.6. Hypothalamic and Hippocampal Samples Preparation

After completion of the behavioral tests and fecal and blood sample preparation, voles were decapitated to study the molecular changes induced by maternal density stress. Following rapid removal of the brain, the entire hypothalamic and hippocampus samples were dissected and immediately frozen in liquid nitrogen and then stored at −80 °C until subsequent analysis.

### 2.7. Hematology Assays

White blood cell counts from stained slides were carried out in the laboratory. Differential white blood cell counts were based on counts of 100 leukocytes.

### 2.8. FCM Assays

The level of circulating corticosterone, which occurred 10–12 h earlier in root voles, can be reflected through fecal corticosterone metabolite level [22]. FCM is primarily derived from plasma-free corticosterone in rodents [23]. FCM measurement is a reliable method to assess individual stress levels in field root vole populations, which has been validated in our previous study [22]. The methods of testing referred to Chen et al. [24].

### 2.9. Cellular Immunity Assays

We first measured the left-hind footpad thickness of root voles with a micrometer before injection (Tesa Shopcal, Urdorf, Switzerland), and then we injected 0.1 mg of PHA (PHA-P, Sigma L-8754, Livonia, MI, USA) dissolved in 0.03 mL sterile saline into the middle of the left footpad. After 6 h, we measured the footpad thickness at the same site. Each measurement was replicated three times on the same individuals. The PHA response was calculated as the difference between pre-injection and post-injection measurements divided by initial footpad thickness [PHA response = (post-PHA − pre-PHA)/pre-PHA] [25].

### 2.10. Humoral Immunity and Cytokines Assays

We used a commercially available mouse anti–KLH IgG ELISA kit (Life Diagnostics Inc., West Chester, PA, USA) to measure the serum anti-KLH IgG level by enzyme-linked immunosorbent assay (ELISA). Each sample was tested in duplicate. The methods of testing referred to Du et al. [15].

The interleukin-1β (IL-1β), interleukin-6 (IL-6), tumor necrosis factor-alpha (TNF-α), and interleukin-10 (IL-10) were then analyzed by ELISA kits (Nanjing Jiancheng Bioengineering Institute, Nanjing, China) using the serum according to the manufacturer’s instructions.

### 2.11. Coccidian Infection Assays

The fecal oocyst counts were determined by a modified McMaster method using a saturated NaCl solution for flotation. More details may refer to our previous research [14]. The prevalence was calculated as the number of infected hosts in enclosures divided by the total number of hosts, which were captured in enclosures. The coccidian intensity of infection was measured as the number of oocysts per gram (OPG) in fresh feces.

### 2.12. DNA Methylation Analysis

Genomic DNA was isolated from hypothalamic and hippocampal samples using the AllPrep DNA/RNA Mini Kit (Qiagen, Duesseldorf, Germany) according to the manufacturer’s instructions, and bisulfite conversion was performed using the EpiTect Fast DNA Bisulfite Kit (Qiagen, Duesseldorf, Germany). Primers (Table S1) for amplifying the bisulfite-converted DNA sequences were designed using PyroMark Assay Design v2.0 software (Qiagen). PCR amplifications were amplified, and the products were separated by agarose electrophoresis and purified by QIAquick Gel Extraction kit (Qiagen, Duesseldorf, Germany). Libraries from different samples were quantified and pooled together equally, sequenced with the Illumina Hiseq 2000 platform according to the manufacturer’s instructions. Analysis of the DNA methylation status of all CpG sites was performed using BSseeker2 software [26].

### 2.13. RNA Extraction, cDNA Synthesis, and RT-qPCR

The RNA isolation from hypothalamic and hippocampal tissues was performed with the aid of Tripure (Roche Applied Science, Penzberg, Germany) following the manufacturer’s protocol. cDNA was synthesized using a HiScript Q RT SuperMix for qPCR kit (Vazyme, R123-01, Nanjing, China) and then analyzed by RT-qPCR using a SYBR Green Real-time PCR Master Mix (Roche, Mannheim, Germany). All reactions were performed in triplicate. For target gene (CRH), 5′-GCCAAGGGAGGAGAAGCAAGC-3′ (forward) and 5′-GCGACAGAGCCATCAACAGGAT-3′ (reverse); (*NR3C1*), 5′-GAGCAGTGGAAGGTAGACAGCAC-3′ (forward) and 5′-AGCTCCTGCAGTGGCTTGCT-3′ (reverse); and for the reference (GAPDH) gene 5′-CCAGAGACACGATGGTGAAGGTC-3′ (forward) and 5′-GTAGTTGAGGTCGATGAAAGGGTCA-3′ (reverse) primer sets were designed. For relative quantification among the results, target gene Ct values were normalized with GAPDH, and the relative fold change in expression was calculated using the 2^−△△ct^ method [27].

### 2.14. Western Blotting

The hypothalamus and hippocampus were homogenized using cold RIPA lysis buffer (G2002-100ML; Servicebio, Wuhan, China). After centrifugation of homogenates, supernatants were collected, and the total protein concentration was determined using a protein assay kit (G2026-200T; Servicebio, Wuhan, China) following the manufacturer’s procedure. Proteins were isolated on 10% polyacrylamide SDS gel and transferred onto a nitrocellulose membrane. The membrane was stained with primary antibodies for CRH (GB11706, dilution 1-1000; Wuhan Servicebio Technology Co., Ltd., Wuhan, China, 2024), *NR3C1* (GB11296, dilution 1-1000; Wuhan Servicebio Technology Co., Ltd., Wuhan, China, 2024) and β Actin (GB11001, dilution 1-2000; Wuhan Servicebio Technology Co., Ltd., Wuhan, China, 2024) (as an internal control), rinsed with TBST (0.1% Tween-20 in TBS) three times for 10 min and then incubated with HRP-conjugated goat-anti-rabbit IgG secondary antibody (GB23303, dilution 1-3000; Wuhan Servicebio Technology Co., Ltd., Wuhan, China, 2024) for 2 h at room temperature. Membranes were developed with Chemi luminescence system ECL kit (G2161-200ML; Wuhan, China) and then exposed to X-ray film. The scanned film signals were quantified and analyzed using AIWBwellTM v.1.0.1 software (Wuhan Servicebio Technology Co., Ltd., Wuhan, China).

### 2.15. Statistical Analysis

Data analyses were performed using SPSS v.22 (IBM, Armonk, NY, USA) software. The data are expressed as the mean ± standard error of the mean (SEM). Hematology parameters (Poisson distribution) and coccidia prevalence (binomial distribution) were analyzed using a generalized linear mixed model (GLMM) with Log/Logit link functions. The differences in PHA response, anti-KLH IgG levels, cytokines levels, the coccidian intensity of infection, the mean DNA methylation, mRNA expression, and protein expression in CRH and *NR3C1* were assessed with the linear model in GLMM between different density groups. The data on the coccidian intensity of infection were ln[OPG + 1] transformed prior to the analyses. Treatments were entered in all the models as fixed factors and enclosure as a random factor. We used repeated measures in GLMM to analyze variations in FCM levels in F1. For repeated measures in GLMM, since response variables may be correlated to the experimental units of study (enclosures) at different time points (trapping session), we first carried out a comparison of candidate models with various covariance structures using the corrected Akaike information criterion. An assessment of the statistical significances was carried out using the sequential Bonferroni post hoc procedure, with Bonferroni corrections at *p* < 0.05.

## 3. Results

### 3.1. F1 FCM Levels and FCM Response to Acute Immobilization Stress

**Figure 1:**
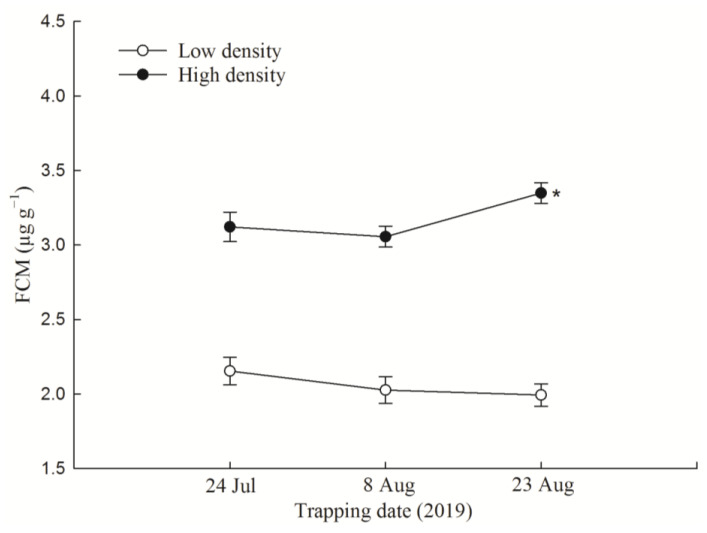
Fecal corticosterone metabolite (FCM) levels across live-trapping sessions in high- and low-density F1. Data were expressed as mean ± SE for each group. “*”, *p* < 0.05 vs. the low-density group. N = 93 and 117 for FCM measurements in low-density and high-density F1 group, respectively.

Mean F1 FCM levels were 2.052 ± 0.049 and 3.158 ± 0.046 μg g^−1^ in low-density and high-density treatment groups, respectively. We found significant effects of treatment (*p* < 0.001) and time × treatment interaction (*p* = 0.023), but no significant effect of time (*p* = 0.179; Figure 1). Statistical analysis indicated that FCM in the high-density treatment group was significantly higher than that in the low-density treatment group across all trapping sessions (*p* < 0.05).

**Figure 2:**
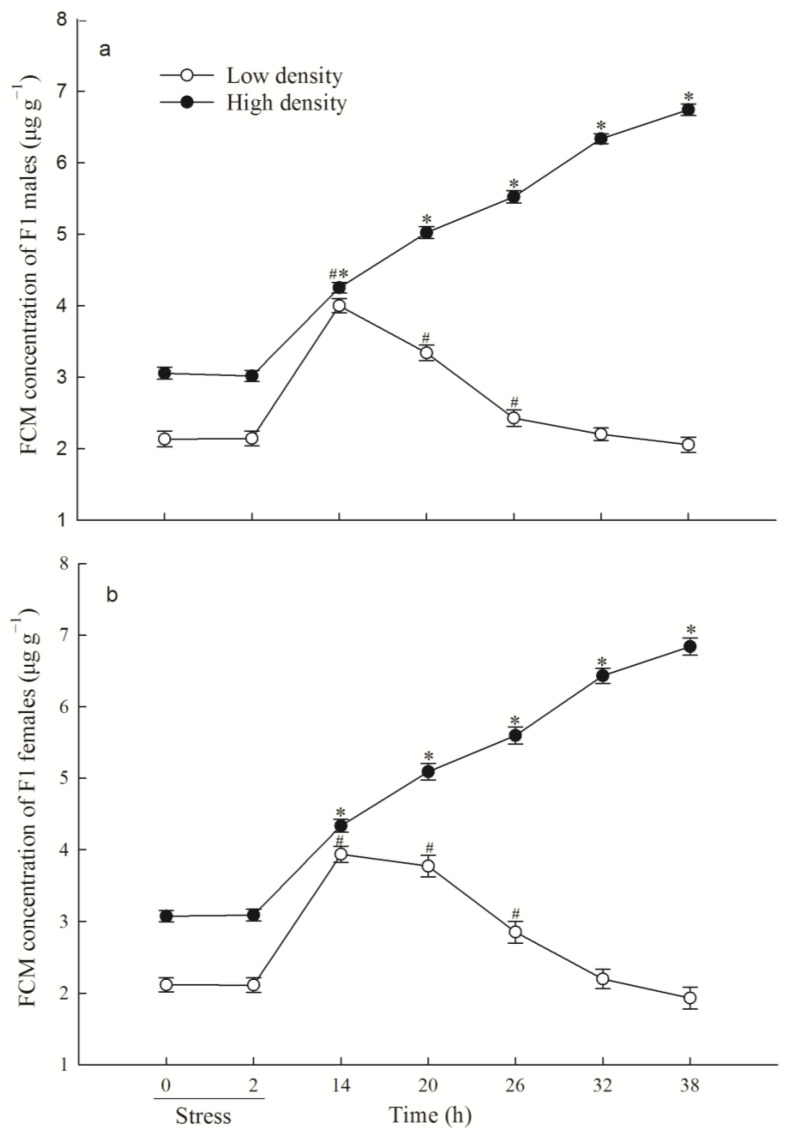
Fecal corticosterone metabolite (FCM) response to acute immobilization stress between two replicate treatment male (**a**) (mean ± SE) (N = 19 and 38 in the low-density and high-density F1 group, respectively) and female (**b**) (mean ± SE) (N = 22 and 36 in the low-density and high-density F1 group, respectively) F1 groups. *p* < 0.05 vs. basal level for the low-density (hash symbol) and high-density groups (asterisk).

When F1 voles were exposed to an acute stressor, those from high-density treatment group significantly increased FCM level at 14 h time point compared with their basal level (14 vs. 0 h, males: *p* < 0.001; females; *p* < 0.001; Figure 2a,b) and still retained the elevated level of FCM at the end of this experiment (38 vs. 0 h, males: *p* < 0.001; females: *p* < 0.001), whereas those from low-density treatment group significantly increased FCM concentration at 14 h (14 vs. 0 h, males: *p* < 0.001; females: *p* < 0.001) and returned to basal values at 32 h ( 32 vs. 0 h, males: *p* = 0.387; females: *p* = 0.166; Figure 2a,b). Thus, F1 voles from high-density parental populations exhibited slow and prolonged CORT responses to acute stress compared with those in the low-density treatment group.

### 3.2. DNA Methylation Levels of CRH Gene in the Hypothalamus and NR3C1 in the Hippocampus

**Figure 3:**
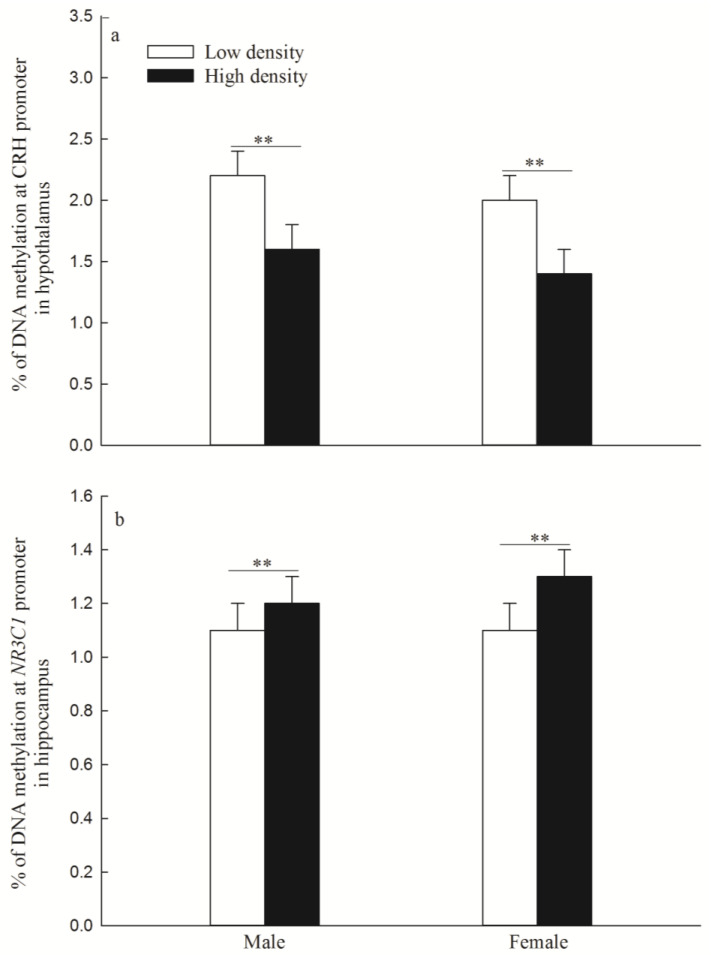
DNA methylation levels of corticotropin-releasing hormone (CRH) gene (**a**) (mean ± SE) and glucocorticoid receptor gene (*NR3C1*) (**b**) (mean ± SE). “**” indicates *p* < 0.01 between low density and high density.

The DNA methylation levels of the CRH gene were significantly lower in the high-density group than in the low-density group (males: *p* = 0.003; females: *p* = 0.001; Figure 3a).

The DNA methylation levels of *NR3C1* were significantly higher in the high-density group than in the low-density group (males: *p* = 0.006; females: *p* = 0.002; Figure 3b).

### 3.3. mRNA and Protein Expression of CRH Gene in the Hypothalamus and NR3C1 in the Hippocampus

**Figure 4:**
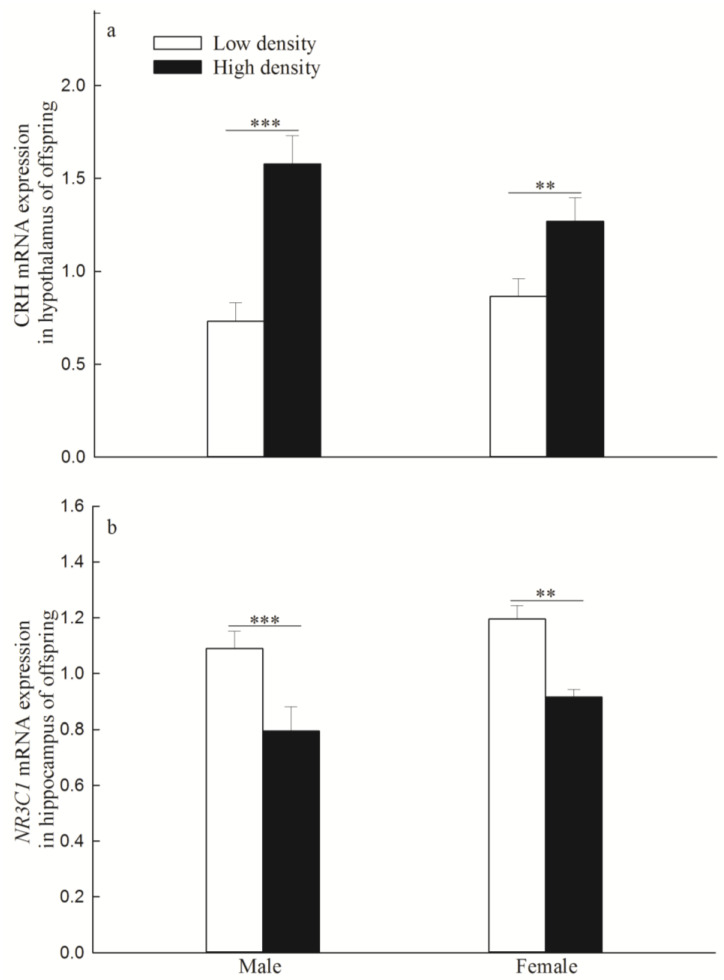
The mRNA expression of corticotropin-releasing hormone (CRH) gene (**a**) (mean ± SE) and glucocorticoid receptor gene (*NR3C1*) (**b**) (mean ± SE). “**” and “***” indicate *p* < 0.01 and *p* < 0.001 between low density and high density, respectively.

The expression levels of the CRH gene mRNA were significantly higher in the high-density group than in the low-density group (males: *p* < 0.001; females: *p* = 0.008; Figure 4a), and the expression levels of the *NR3C1* mRNA were significantly lower in the high-density group than in the low-density group (males: *p* < 0.001; females: *p* = 0.001; Figure 4b).

**Figure 5:**
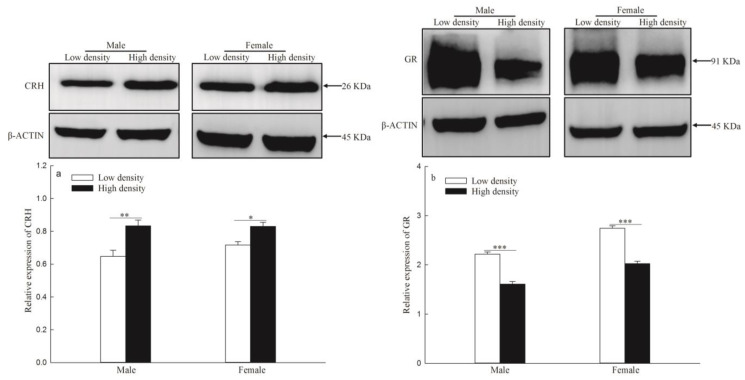
The protein expression of corticotropin-releasing hormone (CRH) (**a**) and glucocorticoid receptor gene (*NR3C1*) (**b**) in F1. Data were expressed as mean ± SE for each group. “*”, “**” and “***” indicate *p* < 0.05, *p* < 0.01 and *p* < 0.001 between low density and high density, respectively.

The levels of CRH gene protein expression of F1 were significantly higher in the high-density group than in the low-density group (males: *p* = 0.002; females: *p* = 0.029; Figure 5a). The levels of *NR3C1* protein expression of F1 showed significantly lower in the high-density group than in the low-density group (males: *p* < 0.001; females: *p* < 0.001; Figure 5b).

### 3.4. Behavioral Tests

**Figure 6:**
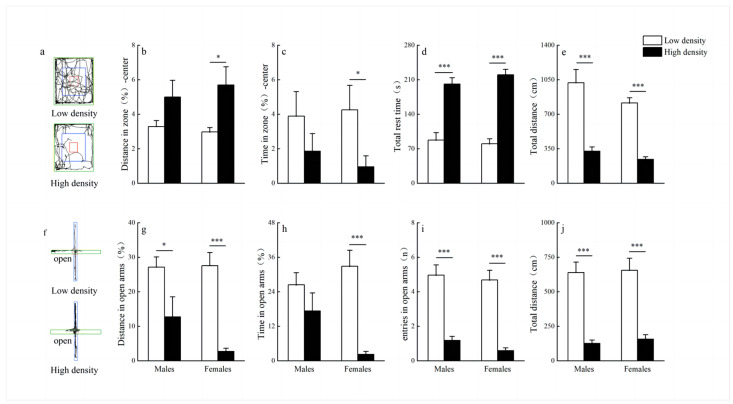
The activity trajectory and activity of root voles in the open field (**a**–**e**) (mean ± SE) (male: N = 20 and 27, female: N = 25 and 29, in the low-density and high-density F1 group, respectively) and the elevated plus maze (**f**–**j**) (mean ± SE) (male: N = 25 and 28, female: N = 19 and 27, in the low-density and high-density F1 group, respectively). “*” and “***” indicate *p* < 0.05 and *p* < 0.001 between low density and high density, respectively.

We used the OFT and EPM to evaluate the effects of maternal density stress on depressive and anxiety-like behavior in F1. In the open field test, the high-density treatment groups had a longer percentage of distance and shorter percentage of time in the center zone in females compared to low-density treatment groups (distance: *p* = 0.022; Figure 6b; time: *p* = 0.029; Figure 6c), while there were no significant differences of the percentage of distance and time in the center zone in males between high-density treatment groups and low-density treatment groups (distance: *p* = 0.153; Figure 6b; time: *p* = 0.236; Figure 6c). In addition, the longer total rest time (males: *p* < 0.001; females: *p* < 0.001; Figure 6d) and shorter total distance (males: *p* < 0.001; females: *p* < 0.001; Figure 6e) were also found in the high-density treatment groups.

In the EPM test, our findings revealed that the high-density treatment groups had a lower percentage of distance in the open arms (males: *p* = 0.038; females: *p* < 0.001; Figure 6g), lower percentage of time in the open arms in females (*p* < 0.001; Figure 6h), lower entries into the open arms (males: *p* < 0.001; females: *p* < 0.001; Figure 6i) and shorter total distance (males: *p* < 0.001; females: *p* < 0.001; Figure 6j) compared to low-density treatment groups.

### 3.5. Inflammatory Cytokines Levels

**Figure 7:**
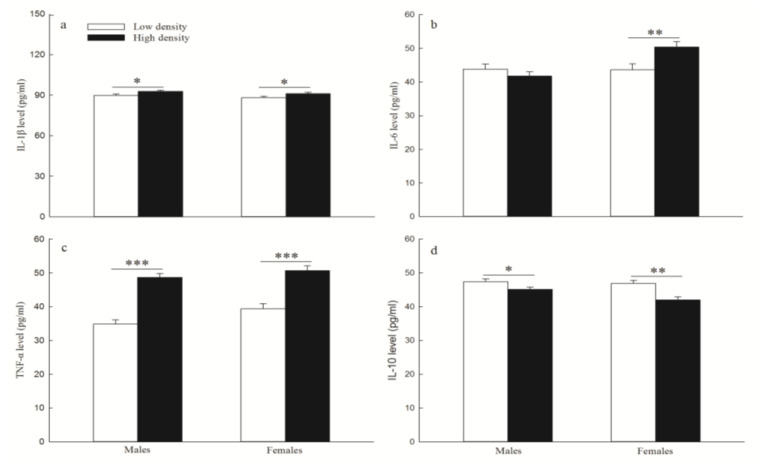
The levels of IL-1β (**a**) (mean ± SE), IL-6 (**b**) (mean ± SE), TNF-α (**c**) (mean ± SE), and IL-10 (**d**) (mean ± SE) between two replicate treatment F1 groups. “*”, “**” and “***” indicate *p* < 0.05, *p* < 0.01 and *p* < 0.001 between low density and high density, respectively. N = 26 and 32 for males, N = 18 and 22 for females, in the low-density and high-density F1 group, respectively.

The high-density treatment group had higher levels of IL-1β (males: *p* = 0.014; females: *p* = 0.030; Figure 7a), IL-6 (females: *p* = 0.007; Figure 7b), and TNF-α (males: *p* < 0.001; females: *p* < 0.001; Figure 7c) compared to low-density treatment groups in F1, except for the level of IL-6 in males (*p* = 0.301). However, the level of IL-10 was significantly lower in the high-density group than in the low-density group (males: *p* = 0.032; females: *p* < 0.001; Figure 7d).

### 3.6. Immune Traits

**Figure 8:**
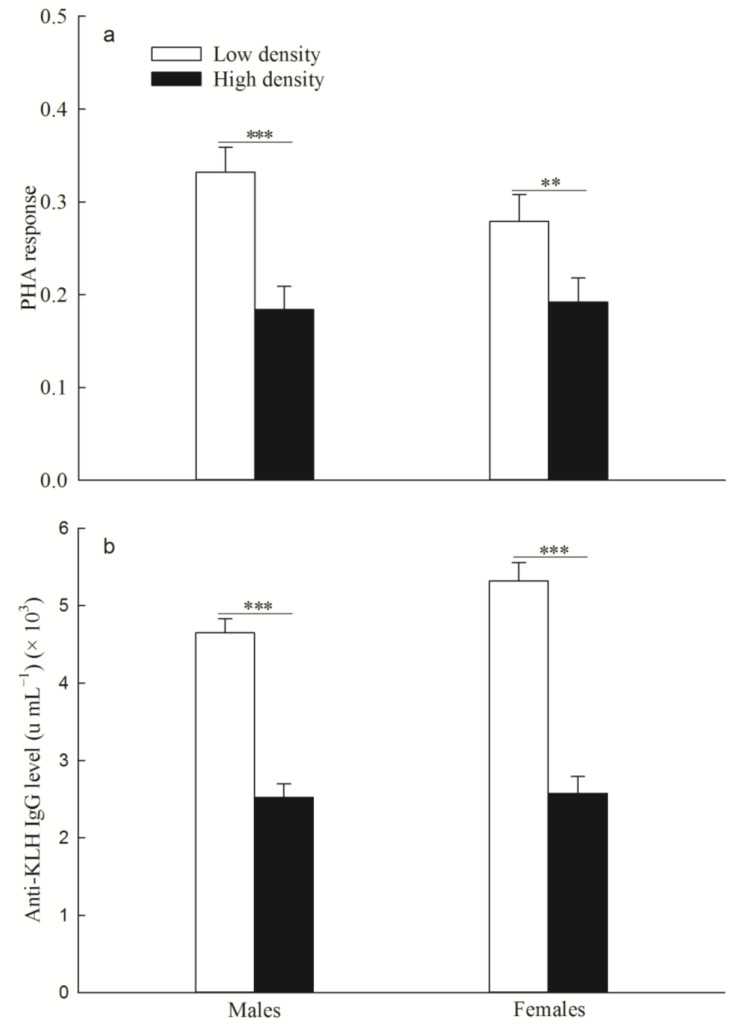
Phytohemagglutinin (PHA) response (**a**) (mean ± SE) (male: N = 23 and 33, female: N = 20 and 28, in the low−density and high−density F1 group, respectively) and anti−keyhole limpet hemocyanin immunoglobulin G (anti−KLH IgG) level (**b**) (mean ± SE) (male: N = 32 and 35, female: N = 19 and 23, in the low−density and high−density F1 group, respectively) between two replicate treatment F1 groups. “**” and “***” indicate *p* < 0.01 and *p* < 0.001 between low density and high density, respectively.

The PHA response (males: *p* < 0.001; females: *p* = 0.026; Figure 8a) and the anti-KLH IgG level (males: *p* < 0.001; females: *p* < 0.001; Figure 8b) were significantly lower in the high-density group than in the low-density group.

Counts of leukocytes (males: *p* = 0.035; females: *p* = 0.025; Table 1) and lymphocytes (males: *p* < 0.001; females: *p* = 0.001; Table 1) were significantly lower in the high-density group than in the low-density group. However, the counts of monocytes (males: *p* = 0.330; females: *p* = 0.650) and granulocytes (males: *p* = 0.677; females: *p* = 0.740) had no significant effect between the high-density group and the low-density group. In addition, F1 plasma protein levels were significantly lower in the high-density treatment group than in the low-density treatment group (males: *p* < 0.001; females: *p* = 0.004; Table 1).

### 3.7. Coccidial Infection

**Figure 9:**
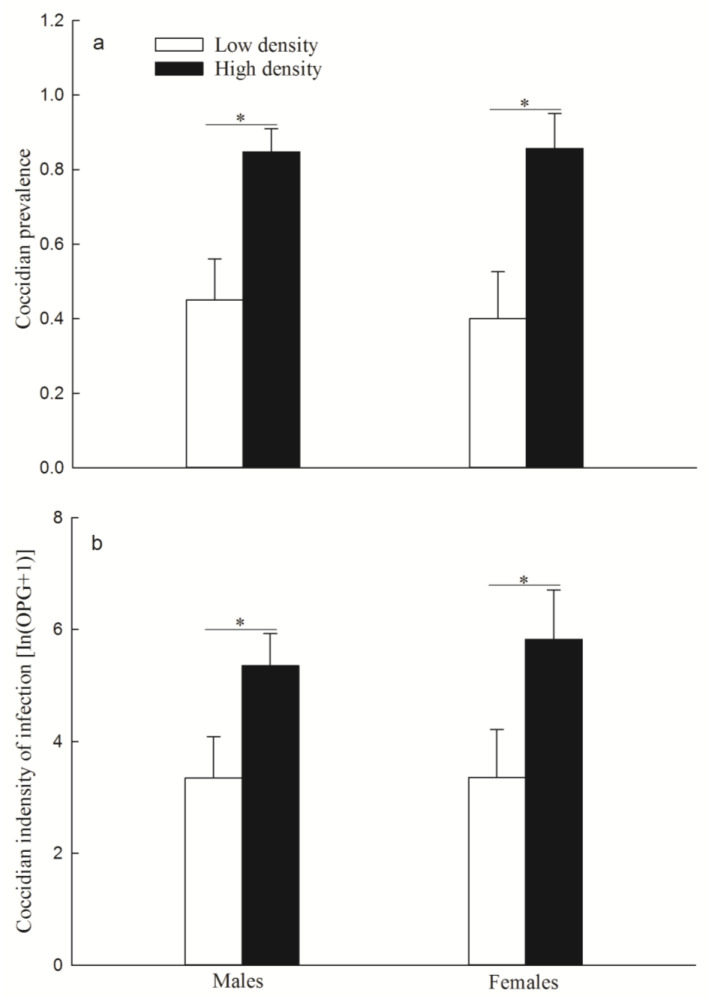
Coccidial prevalence (**a**) (mean ± SE) and intensity of infection (**b**) (mean ± SE) in two replicate F1 groups. * Indicates *p* < 0.05 between low density and high density. N = 20 and 33 for males, N = 15 and 14 for females, in the low-density and high-density F1 group, respectively.

The prevalence of coccidia (males: *p* = 0.035; females: *p* = 0.044; Figure 9a) and the intensity of coccidial infection (males: *p* = 0.035; females: *p* = 0.047; Figure 9b) were significantly higher in the high-density group than in the low-density group.

## 4. Discussion

The effects of DNA methylation of HPA-axis genes of F1 induced by maternal density stress on behavior and immune traits in root voles may be an important direction in regulating population dynamics. In this study, we found that the F1 from the high-density population significantly exhibited slow and prolonged FCM responses to acute immobilization stress and continuously elevated FCM levels, accompanied by lower DNA methylation levels of CRH and higher DNA methylation levels of *NR3C1*, which resulted in an increase in the levels of the CRH mRNA and protein expression, and ultimately reduced the levels of the *NR3C1* mRNA and protein expression (Figure 1, Figure 2, Figure 3, Figure 4 and Figure 5). We also found that juvenile voles born at high density significantly reduced anti-KLH IgG levels and PHA responses (Figure 8), increased cytokines and depressive and anxiety-like behaviors (Figure 6 and Figure 7), and finally led to higher coccidial infection (Figure 9).

Our findings provide a potential mechanism for intrinsic regulation based on epigenetic and expression changes in HPA-axis genes. The adaptive changes of chronic stress are closely related to the HPA axis [3]. The HPA axis, which plays a central role in maintaining physiological stability, can regulate physiological and behavioral changes to enable organisms to adapt to environmental changes [3]. Maternal stress during pregnancy can secrete glucocorticoids, which then reach the fetal brain through placental blood, thereby affecting the development of the fetal immune system [28]. Recent evidence indicates that DNA methylation can mediate the translation of social experiences into enduring alterations in gene expression and the emergence of distinct phenotypes. This form of ‘molecular plasticity’ is believed to enhance an organism’s ability to orchestrate an adaptive response by integrating complex gene-environment interactions [29,30]. Previous studies showed that paternal stress could influence F1 HPA stress system development through epigenetic mechanisms [31,32]. One potential mechanism by which early life stress may act is through programming of the HPA axis and DNA methylation of HPA-axis genes [33,34,35]. However, the above studies are all focused on the study of rats or mice in the laboratory. In our study, F1 in the maternal high-density treatments significantly exhibited slow and prolonged FCM responses to acute immobilization stress and continuously elevated FCM levels, accompanied by lower DNA methylation levels of CRH gene and higher DNA methylation levels of *NR3C1*, which resulted in an increase in the expression levels of the CRH gene mRNA and protein expression, and ultimately reduced the expression levels of the *NR3C1* mRNA and protein expression (Figure 1, Figure 2, Figure 3, Figure 4 and Figure 5). Our results provide new evidence for the effect of maternal density stress on the methylation of HPA axis-related genes in F1 in wild animals.

Continuous stress can have long-lasting adverse effects on behavior, especially in F1 [36]. Mood-related illnesses, including major depression and anxiety, have been associated with negative health outcomes [37]. The hypothalamus and hippocampus are key brain regions involved in emotional disorders and are closely related to the occurrence and recovery of depression and anxiety [16,38]. Studies have shown that prenatal stress leads to a decrease in the total distance of F1 in the open field test and a decrease in the entries in open arms in the elevated plus maze test, which are considered to exhibit depression and anxiety caused by HPA axis dysfunction [39,40]. Previous studies have shown that both chronic and acute stress have been associated with depression and anxiety in both adult and early-life populations [41,42]. In addition, immobilization (IMO) stress or socially isolated prairie voles exhibit increased corticosterone levels and spend less time in the center of the OFT, which suggests that social stress induces HPA axis dysfunction, depressive-like and anxiety-like behavior in the prairie vole [43,44]. However, the above studies on the effects of maternal stress on depression and anxiety behaviors of F1 have mainly been performed in laboratory animals. In our study, we found that F1 in the maternal high-density treatments showed a longer total rest time and shorter total distance in the open field test, a lower percentage of distance in the open arms, lower entries into the open arms, and shorter total distance in the elevated plus maze test accompanied by abnormal function of the HPA axis (Figure 2 and Figure 6). Collectively, we report the new evidence in the wild environment that maternal density stress results in depression and anxiety behaviors caused by abnormal function of the HPA axis in root voles of F1, which involves DNA methylation of HPA-axis genes.

The expression of inflammatory cytokines is also linked to neuropsychiatric diseases and immune response [45,46]. The activation of the HPA axis leads to the secretion of CRH, ACTH, and corticosterone, which modulates the immune response. Conversely, immunity-related substances such as IL-1β, IL-6, and TNF-α can stimulate the HPA axis [47]. Furthermore, the elevated levels of chronic stress lead to a significant increase in pro-inflammatory cytokines such as TNF-α, IL-1β, and IL-6, potentially contributing to neurotransmitter imbalance and alterations in brain regions associated with emotional regulation. Subsequently, this may precipitate mental illnesses, including depression and anxiety disorders [48]. Studies have shown that prenatal stress can significantly increase levels of corticosterone and brain inflammatory cytokines, thereby altering the immune response [45,49]. In prenatally stressed animals, increased levels of IL-1β and TNF-α might influence brain development and stress-related anxiety and depression-like behaviors [50,51]. Prenatal psychological stress can increase maternal inflammatory cytokine levels, resulting in vulnerability to infection in F1 [52]. In our study, we found that F1 in the maternal high-density treatments significantly increased the levels of IL-1β, IL-6, and TNF-α, reduced the level of IL-10 (Figure 7), accompanied by higher depressive and anxiety-like behaviors (Figure 6), lower immunity (Figure 8) and higher coccidial infection (Figure 9), which were in agreement with the above findings.

The immune responses of wild rodents are of great importance to their fitness. There are notably few studies of the immune responses of wild rodents, whereas laboratory studies abound (see review by Viney et al. [53]). Previous studies have indicated that the spleen index and serum IgG levels at high density were significantly higher than those at low density in short-day conditions in male prairie voles [54]. High and medium housing densities in male and female Brandt’s voles also showed significantly higher serum IgG levels [55]. However, Saino et al. [56] found that population density had no significant effects on humoral immunity in bank voles. Thus, studies on immune function impaired by population density are still lacking in wild mammal populations. Wu et al. [57] found that F1 from maternal high-density populations had lower anti-KLH IgG levels and greater spleen weight compared with those from low-density populations of root vole. Although our previous study showed that maternally stressed F1 decreased their anti-KLH IgG level and PHA response [20], the study could not yield a direct result. In the present study, we found that F1 in the maternal high-density group had a lower humoral immune response and cell-mediated immune response compared with those in the maternal low-density group (Figure 8), supporting the above findings and providing a more direct field experimental evidence that maternal density stress can influence immune traits of F1.

Leukocytes play an important role in individual innate immunity and anti-infection [58]. Boonstra et al. [59] found that predation stress significantly reduced the counts of neutrophils, lymphocytes, and monocytes from snowshoe hares. In our study, we found significantly lower counts of leucocytes and lymphocytes in F1 from maternal high-density stress (Table 1). This result was in agreement with that of Boonstra et al. [59].

Coccidian infection is harmful to the wild animals’ health. Coccidian infection not only results in host malnutrition but also wasting [60]. The nutritional status of individuals was closely related to their immune function; for example, individuals with lowered nutrition had accompanying lowered immune functions [61]. Some studies have suggested that coccidian parasites may seriously affect the host’s nutrition absorption, leading to a decline in immune function [62,63]. Our previous studies have shown that parasitic infection had a significantly inhibitory effect on cellular immunity and humoral immunity [15,20,64]. In the present study, maternally stressed F1 had higher coccidian infection (prevalence and intensity of infection), lower plasma protein level, and eventually lower immunity (Figure 8 and Figure 9; Table 1), which were in agreement with the above studies.

## 5. Conclusions

In summary, the present study provides new evidence in wild F1 populations that maternal density stress results in higher depressive and anxiety-like behaviors, higher levels of inflammatory cytokines, higher coccidial infection, and lower immune traits caused by abnormal function of the HPA axis, which involved DNA methylation of HPA-axis genes. Our results suggested that DNA methylation differences are a fundamentally important mechanism that can affect behavior and immune traits in F1 in wild animals. This, in turn, can drive population cycles from the perspective of epigenetics in wild animals. Future studies aiming at verifying the interactions between intrinsic and extrinsic factors from the perspective of epigenetics, which can be important determinants of population decline, would be necessary.

## Figures and Tables

**Table 1:** Hematology characteristics [mean ± SE(n)] of root voles in F1.

Parameters	Low Density		High Density	
	Male	Female	Male	Female
White blood cell (×10^9^)	5.43 ± 0.59 (32) *	5.59 ± 0.61 (19) ^#^	3.89 ± 0.42 (35)	3.91 ± 0.42 (23)
Lymphocytes (×10^9^)	3.20 ± 0.22 (32) *	3.22 ± 0.22 (19) ^#^	2.22 ± 0.15 (35)	2.25 ± 0.16 (23)
Monocytes (×10^9^)	0.62 ± 0.04 (32)	0.59 ± 0.04 (19)	0.57 ± 0.04 (35)	0.62 ± 0.04 (23)
Granulocytes (×10^9^)	0.18 ± 0.37 (32)	0.18 ± 0.37 (19)	0.16 ± 0.33 (35)	0.17 ± 0.34 (23)
Plasma proteins (mg mL^−1^)	10.32 ± 1.22 (32) *	9.26 ± 1.33 (19) ^#^	3.87 ± 1.20 (35)	3.77 ± 1.27(23)

* Indicates a statistically significant difference between low density and high density in male F1. # Indicates statistically a significant difference between low density and high density in female F1.

## Data Availability

The data used in this study are available from the corresponding author on request.

## Supplementary Materials

The following supporting information can be downloaded at: https://www.mdpi.com/article/10.3390/ani14172467/s1. Table S1: Primers for amplifying the bisulfite-converted DNA sequences in CRH and *NR3C1* gene.
