# Supplementary material for: Effects of DNA Methylation of HPA-Axis Genes of F1 Juvenile Induced by Maternal Density Stress on Behavior and Immune Traits in Root Voles (Microtus oeconomus)—A Field Experiment
Source: Animals (Basel). 2024 Aug 25;14(17):2467. doi: 10.3390/ani14172467 (PMC11393846; doi:10.3390/ani14172467)
Supplement: Supplementary file 1 [file animals-14-02467-s001.zip › animals-3142857-supplementary-table s1.pdf]

Table S1 Primers for amplifying the bisulfite-converted DNA sequences in CRH and NR3C1gene

Target	Gene	Primer F	Primer R
CRH_1	CRH	TATTTTGATTYGTATGGGAGAAGAATA	AACRCTAAAACRTCTACAACTACTACAACA
CRH_2	CRH	TGTTGTAGTAGTTGTAGAYGTTTTAGYGTT	CTAACTACTCTACCCTAACCATTTCCA
NR3C1_1	NR3C1	GGGTTTTAAGAYGGTTTAGGTTTAGTTT	ACCTCCCRCCRCCATCTTAA
NR3C1_10	NR3C1	GGGTTTTGGGTTGTTTTTGG	TATATCCAAACCTATTAAATTCTCTACRACAC
NR3C1_11	NR3C1	GAGGGTTTGAGGTAAAAAGTAAAAT	ACTTAACAAACTTTTATCAACCCTAAAAT
NR3C1_12	NR3C1	ATTTTAGGGTTGATAAAAGTTTGTTAAGT	ACTCTCCCCTCCCCCATAAA
NR3C1_13	NR3C1	GAGTTYGAGTTAGTGTTTGGAGTTTTAGT	CAACAATCRCCCCCTCTTCTCCATA
NR3C1_2	NR3C1	TTAAGATGGYGGYGGGAGGT	AATACCCCCACAAATAACAACACTT
NR3C1_3	NR3C1	AAGTGTTGTTATTTGTGGGGGTATT	TCCCAAAAAAACACRTAACCAATC
NR3C1_4	NR3C1	GATTGGTTAYGTGTTTTTTTGGGA	CCAACCCRCAAAATTCCAAAAAC
NR3C1_5	NR3C1	GTGAGTTYGTGGGGGGGATT	AATAAATTCTACTTTACAACTTCTCTCRATAC
NR3C1_6	NR3C1	GTATYGAGAGAAGTTGTAAAGTAGAATTTATT	TTTTTCAAATACAAAAAAAACCTACTC
NR3C1_7	NR3C1	AGTTTTTTTTAGAGGGYGTGTTTGT	TTTCTCTTCTCCCTAACTCCTTCC
NR3C1_8	NR3C1	GGAAGGAGTTAGGGAGAAGAGAAA	CCAACCCRACCCCRACRAC
NR3C1_9	NR3C1	TGTGTATTAGTTTTGGGGTAYGATTT	ACATTTATCCTCCAATTAAAACTATAAATAACC
